# Newborn Vitals in the First 10 min Do Not Differ With Mode of Delivery

**DOI:** 10.1155/ijpe/7764047

**Published:** 2026-04-11

**Authors:** Archana S. Nimbalkar, Minal Patel, Krishna Mori, Shraddha Patel, Reshma Pujara, Dipen V. Patel, Mayur K. Shinde, Somashekhar M. Nimbalkar

**Affiliations:** ^1^ Department of Physiology, Pramukhswami Medical College, Bhaikaka University, Karamsad, Gujarat, India, bhaikakauniv.edu.in; ^2^ Department of Pediatrics, Pramukhswami Medical College, Bhaikaka University, Karamsad, Gujarat, India, bhaikakauniv.edu.in; ^3^ Department of Neonatology, Pramukhswami Medical College, Bhaikaka University, Karamsad, Gujarat, India, bhaikakauniv.edu.in; ^4^ Central Research Services, Bhaikaka University, Karamsad, Gujarat, India, bhaikakauniv.edu.in

## Abstract

We compared the physiological transition of neonates > 35 weeks old that are born vaginally versus those born by elective LSCS using SpO2, heart rate, and temperature in the first 10 min of life. We recorded the SpO2 of babies at 0, 15, 30, 45, 60, and 75 s until 5 min (15‐s intervals) and every 30 s after that until 10 min. Heart rate was recorded using a stethoscope at 30‐s intervals by counting for 6 s and multiplying by 10. The Temperature was recorded every 1–10 min via a digital thermometer. The study included 221 neonates, with 111 being born vaginally. Baseline characteristics such as gestational age, sex, and weight were comparable. The mean duration of skin‐to‐skin contact was higher in the vaginal deliveries as compared with LSCS (32.1 [8.6] vs. 4.6 [4], *p* < 0.001). The mean SpO2 in both groups at different time intervals was not statistically significant up to 6 min. It was higher in normal vaginal deliveries until 9 min (statistically significant). After 9 min, it was similar in both groups. Heart rate and temperature were similar in both groups. Physiological parameters in the newborn do not differ much despite the difference in delivery methods.

## 1. Introduction

The newborn transitions from the fetal environment to the external atmospheric environment at birth via a complex process that requires numerous physiological changes. This transition results in various changes across the various organ systems of the newborn, from the respiratory and cardiovascular systems to the neuronal and endocrine organs, too. A smooth transition results in a newborn who is healthy and well adjusted to the new environment [1]. The past few decades have allowed us to study this physiology of transition in greater detail due to the availability of newer pieces of equipment and a large body of work that has happened in animal experiments, allowing us to extrapolate some findings to the human species [2, 3].

The fetus‐to‐newborn physiologic transition begins before delivery in utero. This transition depends on several factors, including the health of the mother, chronic medical conditions, the placenta′s health, duration of gestation, and the presence of fetal anomalies. Thus, the physiology of this transition is complex and requires an understanding of the cardiovascular and pulmonary systems in utero and ex‐utero [4].

The onset of fetal lung fluid clearance initiates the successful transition. At birth, the newborn must clear this liquid rapidly to allow air entry and begin the onset of gas exchange [5]. Clearance of this fluid is possible due to cellular and mechanical mechanisms. Three different mechanisms are likely to be at play. First, the mechanical contractions of the uterus during labor play a vital role by increasing the fetal abdominal pressure and forcing the fetal diaphragm upwards. This increases airway pressure by pushing lung fluid out of the lung via the trachea. The hormone cortisol drives the cellular mechanism. Cortisol production increases significantly at the end of the third trimester due to the maturation of the fetal adrenal gland. Increased cortisol leads to increased surfactant production that, in turn, reduces alveolar surface tension while maintaining alveolar expansion. Cortisol also increases *β*‐adrenergic receptors within the lung, which leads to an increase in the transcription of genes that produce epithelial sodium channels. These channels change the lung from a chloride‐secreting organ into one that reabsorbs sodium. This ensures the absorption of fetal lung fluid from the alveolar air spaces into the interstitium and intravascular spaces. Studies in sheep have shown that this transition begins much before the onset of labor but increases significantly during labor. Bland et al. discovered that sheep delivered after the onset of labor had 45% less lung fluid than those delivered without going through labor [4–6].

Unless severely hypoxic due to secondary apnea, the fetus moves into a pattern of regular spontaneous breathing soon after birth. The neonates who achieve oxygen saturation levels > 90% within the first 10 min of life and show a normal respiratory pattern and respiratory rate within the first 90 min of life can be considered to have made a successful respiratory transition to extrauterine life [7].

Blood oxygen levels are low during fetal development. The mean pO_2_ of a fetus is 25–35 mmHg, whereas within minutes after birth, the normal partial pressure of oxygen (pO_2_) in healthy newborns is 65–75 mmHg. The oxygen saturation increases from ∼55% at birth to ∼85% (pO_2_ 65–75 mmHg) by 5 min and to > 95% by ∼10 min after the onset of breathing. Preterm neonates usually have a more extended transition period and often require up to 10 min to reach SpO_2_ of 85% [8].

Many of the above factors discussed for moving out of the lung fluid into the circulation are not in play during a lower‐segment cesarean section (LSCS). Hence, it is likely that the transition in these babies is different. Prelabor LSCS, deprived of the beneficial hormonal and neuroendocrine advantages of the onset and progress of labor, leads to an increased risk of delayed lung fluid clearance, respiratory distress syndrome, air leak syndrome, and increased risk of neonatal resuscitation, especially if performed before 39 weeks [9]. In one of the first studies that looked at preductal and postductal SpO_2_ in both preterm and term neonates, the SpO_2_ levels were lower in babies born by LSCS vis‐à‐vis those born through the vaginal route. The mean time for the preductal SpO_2_ level to reach 90% was 5.2 min (SD –1.5) in vaginal deliveries, whereas it was 6.3 min (SD –2.4) after LSCS [10].

The rise in heart rate (HR) of the neonate generally parallels the increase in saturation and measurement of HR during the immediate period following birth. Therefore, HR monitoring is of great significance during the transition and has been used for years to manage resuscitation. Various methods are used to determine the measurement of HR, with intermittent contact methods being used for decades. This includes auscultation and palpation, a digital stethoscope, and a handheld Doppler [11].

Most of the focus has been placed on babies around vaginal delivery in studies done at our institution. At the same time, almost 50% of the newborns delivered are born through LSCS. It is well known that LSCS deliveries of term infants and near‐term infants are associated with a high risk of neonatal outcomes. While respiratory distress syndrome and severe hypoxemic failure are seen due to underlying pathologies in the newborn, even relatively low‐risk term babies have a high incidence of transient tachypnea. This is due to the delayed lung fluid clearance, which is intricately linked to the newborn′s physiological transition at birth [12].

The research on neonatal transition has mainly focused on vaginal deliveries worldwide, as it is the most common mode. It is also known that LSCS neonates may have a high risk, and hence, it is expected that they may require substantial assistance to transition after birth. However, as mothers get referred early to hospitals for high‐risk delivery, and with the increased focus on delivering in the hospital over the last one and a half decades, we see more LSCS that are planned. However, the transition of babies born through an LSCS has not been well documented in our region, and hence, we decided to address this area of research.

We compared the physiological transition of neonates > 35 weeks old that are born vaginally versus those born by elective LSCS with the use of three parameters (SpO_2_, HR, and temperature for the first 10 min of life).

## 2. Materials and Methodology

The prospective observational analytical study was conducted at Shree Krishna Hospital between February 2020 and February 2021. We collected data from the delivery room and the operating theater′s newborn resuscitation areas. We approached mothers who were likely to deliver a neonate who was more than 35 weeks old, either by LSCS or by vaginal delivery, and obtained their written consent for the use of their data for analysis. All newborns over 35 weeks old were included in this study. The exclusion criteria included those neonates who needed bag and mask ventilation, those who had obvious life‐threatening congenital malformations, neonates with congenital cardiac diseases (diagnosed antenatally or in the first week of life), supplemental oxygen requirements, medications requirements at birth, neonatal intensive care unit admission, and inability to adequately obtain required data in the first 10 min after birth.

The Department of Pediatrics records SpO_2_, temperature, and HR to document normal transitions per the 2015 NRP guidelines. Although the department regularly records this data, our study ensured a more detailed recording at regular intervals of 0, 15, 30, 45, 60, and 75 s, until 5 min (15‐s intervals) and every 30 s after that, till 10 min using the SpO_2_ that is routinely attached to the newborn′s right wrist. Pulse oximetry was performed using new‐generation pulse oximeters (Masimo Radical, Masimo, Irvine, California, United States). The sensors were placed on the right hand for the preductal SpO_2_ monitoring. The maximum sensitivity setting was used for measurement. HR was monitored using a stethoscope at 30‐s intervals by counting 6 s and multiplying by 10. The Temperature was recorded every minute for 10 min via a digital thermometer in the labor room. In addition, we obtained demographic data from patient records for further analysis.

The babies born via the normal vaginal route were placed prone on the mother′s abdomen and dried as per the 2015 guidelines. The newborn remained there until the mother was moved to the postnatal ward. Then, the baby was removed from the abdomen and placed back on the abdomen when the mother reached the postnatal ward (usually within 1–2 min).

The babies born via LSCS were placed on a warmer and shifted to the mother′s side after an hour. Their recording was done similarly to the vaginal deliveries.

In both sets of babies, delayed umbilical cord clamping was practiced for about 1 min of life, as it has been a routine practice since 2015 after the change of guidelines.

Time of birth was considered when the last fetal part was out in both normal vaginal delivery and LSCS.

The institutional Ethics Committee approved the study. STATA was used to analyze the data using descriptive statistics (mean [SD], frequency (%), etc). We used the independent sample *t*‐test to evaluate the change in mean SpO_2_, HR, and Temperature at each time point.

## 3. Results

The study included 221 neonates (53 females and 58 males in the normal vaginal delivery group and 44 females and 66 males in the LSCS group) from the obstetrics department of Shree Krishna Hospital, Karamsad, who fulfilled the inclusion and exclusion criteria. Of these, 111 were from normal vaginal delivery (Group NVD), and the rest were from group LSCS.

**Figure 1:**
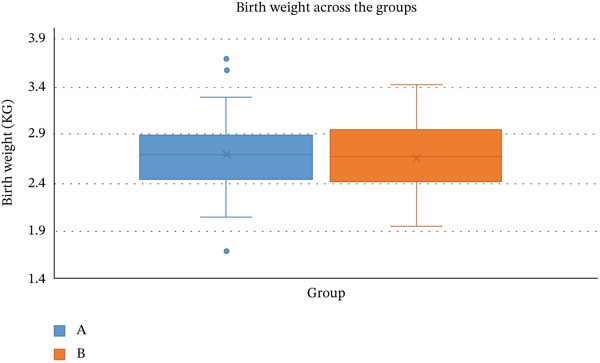
Box plot showing the distribution of the birth weight of neonates.

The mean (SD) birthweight in Group A (NVD) and Group B (LSCS) was (2.69 [0.33] vs. 2.67 [0.36], *p* = 0.67). The box plot in Figure 1 shows that the birthweight distribution was almost similar and comparable (*p* > 0.05) in both NVD and LSCS.

The gestational age mean (SD) was around (38.12 [1.28] vs. 37.89 [1.39]) weeks in Group A (NVD) and Group B (LSCS). Independent sample *t*‐test showed that there was no statistically significant difference in the groups (*p* = 0.19).

Both groups have comparable baseline characteristics of mothers, except for parity, abortion, and live birth, as shown in Table 1. The proportion of the mothers who had a single abortion and two or more abortions in Group A (NVD) was 17.1%, and 2.7% versus in Group B (LSCS) was 18.1% and 11.8%, which was statistically significant (*p* = 0.002). The proportion of the mothers having one, two, three, and more than four live births in Group A (NVD) was 35.1, 35.5, 15.3, and 14.4, whereas in Group B (LSCS), 36.3, 37.2, 17.2, and 2.7, which was also statistically significant (*p* = 0.03). Also, the proportion of the parity in both groups was not comparable (*p* < 0.005) (Table 1).

**Table 1:** Comparison of baseline characteristics of the mother in both groups.

Parameters		Group A (NVD) *n* = 111(%) *n* (%)	Group B (LSCS) *n* = 110(%) *n* (%)	*p*
Small for gestational age/appropriate for gestational age	Appropriate for gestational age	90 (81.08)	88 (80)	0.83
	Small for gestational age	21 (18.92)	22 (20)	
Gravida	1	35 (31.53)	28 (25.45)	0.06
	2	31 (27.93)	40 (36.36)	
	3	19 (17.12)	28 (25.45)	
	≥4	26 (23.42)	14 (12.73)	
Parity in pregnancy	0	3 (2.70)	0 (0.00)	0.005
	1	38 (34.23)	36 (32.73)	
	2	36 (32.43)	47 (42.73)	
	3	16 (14.41)	23 (20.91)	
	≥ 4	18 (16.22)	4 (3.64)	
Abortion	0	89 (80.18)	77 (70.00)	0.02
	1	19 (17.12)	20 (18.18)	
	≥ 2	3 (2.70)	13 (11.82)	
Live birth	0	4 (3.60)	7 (6.36)	0.03
	1	39 (35.14)	40 (36.36)	
	2	35 (31.53)	41 (37.27)	
	3	17 (15.32)	19 (17.27)	
	≥ 4	16 (14.41)	3 (2.73)	
Maternal hypertension	Yes	10 (9.01)	14 (12.73)	0.37
	No	101 (90.99)	96 (87.27)	
Fetal distress	Yes	0 (0)	2 (1.82)	0.24

The mean (SD) duration of the skin‐to‐skin contact in Group A (NVD) and Group B LSCS) was (32.1 [8.6] vs. 4.6 [4], p <0.001). Table 2 depicts the distribution of the duration of skin‐to‐skin contact (minutes), which was statistically significant (*p* < 0.001).

**Table 2:** Distribution of the skin‐to‐skin contact and room temperature in both groups.

Parameters	Group A (NVD) mean (SD)	Group B (LSCS) mean (SD)	*p*
Duration of skin‐to‐skin contact (minutes)	32.12 (8.63)	4.6 (4.00)	< 0.001
Room temp	32.08 (0.98)	31.84 (0.88)	0.059

The distribution of the room temperature in both groups was similar and hence comparable, and it was 32.08°C in Group A (NVD) and 31.84°C in Group B (LSCS) (Table 2).

## 4. Physiological Parameters

The mean SpO_2_ in both groups at different time intervals was not statistically significant up to 6 min. However, it was higher in Group A than in Group B from 6 to 9 min, which was statistically significant. After 9 min, both groups were again similar (Table 3). These details are further explained in Table 4 where the saturation blocks and different time‐points are shown.

**Table 3:** Saturation readings of the groups showing detailed data.

Time in seconds	Group A (NVD) *n* = 111 mean (SD)	Group B (LSCS) *n* = 110 mean (SD)	*p*
0	Not available	Not available	
60 (*A* = 45 and *B* = 43)	77.46 (1.28)	77.32 (1.45)	0.632
75	79.24 (1.32)	79.3 (1.51)	0.767
90	79.92(1.30)	79.89 (1.47)	0.844
105	80.57 (1.33)	80.6 (1.22)	0.892
120	81.20 (1.33)	81.32 (1.25)	0.492
135	81.97 (1.30)	82.00 (1.39)	0.843
150	82.55 (1.57)	82.60 (1.36)	0.799
165	82.88 (4.61)	83.22 (1.78)	0.466
180	84.07 (1.96)	84.19 (2.01)	0.657
195	85.24 (2.41)	85.08 (2.42)	0.620
210	86.09 (2.49)	86.07 (2.66)	0.960
225	87.07 (2.64)	86.85 (2.62)	0.540
240	87.75 (2.57)	87.46 (2.47)	0.390
255	88.63 (2.63)	88.22 (2.58)	0.241
270	90.77 (2.39)	88.87 (2.52)	0.290
285	90.12 (2.26)	89.62 (2.46)	0.119
300	90.90 (2.12)	90.44 (2.34)	0.124
330	91.52 (1.88)	91.03 (2.29)	0.087
360	92.25 (1.79)	91.73 (2.25)	0.061
390	93.00 (1.78)	92.4 (2.05)	0.020
420	93.45 (1.90)	92.9 (1.90)	0.033
450	94.08 (1.79)	93.38 (1.80)	0.004
480	94.56 (1.54)	93.93 (1.64)	0.004
510	95.17 (1.56)	94.63 (1.51)	0.010
540	95.62 (1.38)	95.2 (1.35)	0.023
570	96.07 (1.26)	95.77 (1.28)	0.083
600	96.53 (1.24)	96.40 (1.15)	0.448

**Table 4:** SpO2 between groups at various timepoints. Details as follows:

Time points in seconds	SpO2	Group A (NVD) *n* = 111*n* (%)	Group B (LSCS) *n* = 110*n* (%)	*p*
60 (*A* = 45 and *B* = 43)	74–80	45 (100)	43 (100)	NA
75	74–80	99 (89.19)	92 (83.64)	0.22
	81–85	12 (10.81)	18 (16.36)	
90	74–80	80 (72.07)	66 (60)	0.058
	81–85	31 (27.93)	44 (40)	
120	74–80	28 (25.23)	24 (21.82)	0.58^a^
	81–85	82 (73.87)	86 (78.18)	
	86–90	1 (0.90)	0	
150	74–80	6 (5.41)	5 (4.55)	0.91
	81‐85	99 (89.19)	100 (90.91)	
	86–90	6 (5.41)	5 (4.55)	
180	74–80	1 (0.90)	1 (0.90)	0.97^a^
	81–85	92 (82.88)	90 (81.82)	
	86–90	16 (14.41)	17 (15.45)	
	91–95	2 (1.80)	2 (1.80)	
210	81–85	60 (54.05)	62 (56.36)	0.86
	86–90	40 (36.04)	36 (32.73)	
	91–95	11 (9.91)	12 (10.91)	
240	81–85	18 (16.22)	23 (20.91)	0.64^a^
	86–90	75 (67.57)	70 (63.64)	
	91–95	18 (16.22)	16 (14.55)	
	>95	0	1 (0.91)	
270	81–85	5 (4.50)	5 (4.50)	0.67^a^
	86–90	67 (60.36)	75 (68.18)	
	91–95	38 (34.23)	29 (26.36)	
	> 95	1 (0.90)	1 (0.90)	
300	81–85	0	1 (0.91)	0.14^a^
	86–90	44 (39.64)	56 (50.91)	
	91–95	66 (59.46)	52 (47.27)	
	> 95	1 (0.90)	1 (0.90)	
330	81–85	0	2 (1.82)	0.07^a^
	86–90	33 (29.73)	43 (39.09)	
	91–95	78 (70.27)	64 (58.18)	
	> 95	0	1 (0.91)	
360	81–85	0	1 (0.91)	0.02^a^
	86–90	19 (17.12)	35 (31.82)	
	91–95	90 (81.08)	72 (65.45)	
	> 95	2 (1.82)	2 (1.82)	
390	86–90	12 (10.81)	22 (20.00)	0.163
	91–95	93 (83.78)	82 (74.55)	
	> 95	6 (5.41)	6 (5.41)	
420	86–90	8 (7.21)	10 (9.09)	0.80
	91–95	92 (82.88)	91 (82.73)	
	> 95	11 (9.91)	9 (8.18)	
450	86‐90	39 (2.70)	3 (2.70)	0.70^a^
	91–95	87 (78.38)	91 (82.73)	
	> 95	21 (18.92)	16 (14.55)	
480	86‐90	1 (0.90)	1 (0.90)	0.34^a^
	91–95	79 (71.17)	87 (79.09)	
	>95	31 (27.93)	22 (20)	
510	91–95	57 (51.35)	80 (72.73)	0.001
	> 95	54 (48.65)	30 (27.27)	
540	91–95	49 (44.14)	62 (56.36)	0.069
	> 95	62 (56.36)	48 (43.64)	
570	91–95	35 (31.53)	47 (42.73)	0.08
	> 95	76 (68.47)	63 (57.27)	
600	91–95	20 (18.02)	23 (20.91)	0.58
	> 95	91 (81.98)	87 (79.09)	

^a^Fisher′s exact test.

**Figure 2:**
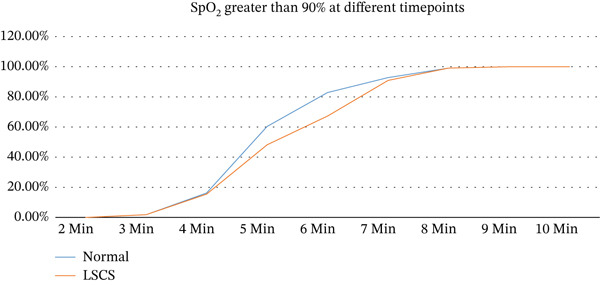
SpO_2_ greater than 90% at different time points across the groups.

Normal delivery had a significantly higher proportion of SpO_2_ in the 91–95 range at 6 min than LSCS (81.08% vs. 65%; *p* = 0.02). Similarly, at 8.5 min, the proportion of neonates whose SpO_2_ was in the range of > 95 was statistically significantly higher in the normally delivered group than in the LSCS group (48.65% vs. 27.27%; *p* = 0.001). These differences are shown in Figure 2.

The line chart in Figure 2 shows that SpO_2_ increased faster in the normal delivery group than in the LSCS group, from 4 to 7 min.

HR was recorded every 30 s after birth for 10 min. There was no statistically significant difference in the HR of the neonates between the two groups. This can be seen in Table 5.

**Table 5:** Heart rate for the two groups over 10 min.

Time in seconds	Group A (NVD) *n* = 111 mean (SD)	Group B (LSCS) *n* = 110 mean (SD)	*p*
0	Not available	Not available	
60	110.71 (4.84)	110.75 (3.89)	0.942
90	113.90 (5.61)	113.60 (5.05)	0.926
120	117.03 (6.21)	116.86 (4.92)	0.819
150	120.09 (6.92)	119.69 (6.82)	0.666
180	123.09 (7.69)	122.92 (7.71)	0.868
210	125.99 (8.78)	126.47 (9.07)	0.689
240	128.51 (8.99)	129.43 (10.28)	0.478
270	131.68 (9.23)	132.69 (10.31)	0.446
300	135.34 (8.98)	136.24 (10.34)	0.489
330	138.35 (9.47)	140.26 (10.61)	0.159
360	142.64 (9.57)	143.76 (9.89)	0.396
390	145.27 (10.11)	146.43 (9.15)	0.370
420	148.63 (10.21)	149.21 (7.46)	0.626
450	151.19 (6.95)	152.12 (7.03)	0.325
480	153.34 (7.33)	155.82 (6.13)	0.007
510	156.96 (6.81)	157.77 (6.09)	0.354
540	159.83 (6.85)	161.36 (6.14)	0.083
570	161.50 (7.32)	162.97 (6.07)	0.106
600	164.90 (6.41)	165.72 (6.16)	0.330

Temperatures were taken every 1 min, immediately after the birth, to 10 min via a digital thermometer in the labor room. Table 6 depicts the mean (SD) of the neonate′s temperature (in °F). No statistically significant difference was found between Group A and Group B.

**Table 6:** Group‐wise temperature transition of neonates.

Time in seconds	Group A (NVD) *n* = 111*n* (SD)	Group B (LSCS) *n* = 110*n* (SD)	*p*
0	Not available	Not available	
60	95.51 (0.34)	95.51 (0.34)	0.971
120	96.65 (0.73)	96.57 (0.72)	0.398
180	96.84 (0.66)	96.79 (0.72)	0.549
240	97.02 (0.60)	97.05 (0.75)	0.734
300	97.21 (0.50)	97.28 (0.65)	0.361
360	97.39 (0.47)	97.46 (0.62)	0.393
420	97.76 (0.48)	97.85 (0.54)	0.197
480	97.96 (0.44)	97.96 (0.46)	0.921
540	98.15 (0.34)	98.10 (0.38)	0.242
600	98.31 (0.24)	98.25 (0.25)	0.068

## 5. Discussion

The current study describes the differences between oxygen saturation and HR in neonates born after normal vaginal delivery or LSCS. We do not find much difference, but previous studies have reported different results. Newborns remain poorly saturated after birth, but their saturation rises slowly with time [13, 14]. The 2010 NRP algorithm used the Dawson curves for the first time to show the slow rise in saturation in term infants based on the study published in 2010 [15]. Studies done early on did not show much difference between LSCS and NVD [16–19]. House et al. placed all 100 babies on a radiant warmer after immediate cord clamping and had a patient population varying from 850 gm to 5230 gm [16]. Isobe et al. had a smaller sample of 20 babies only and found no significant differences too [18]. Dimich et al. also studied 100 neonates but found no differences between the saturation of LSCS or NVD [17] More recently, in a study done in Turkey, 40 neonates were studied across LSCS and NVD, but no significant differences were found [19]. In another study from Qatar, Habboub et al. found no difference between LSCS and NVD. However, the report does not mention the time of transition and measurement, and probably reports SpO_2_ after stabilization, which is not what we studied [20]. In most of these studies, the oxygen saturation was independent of APGAR score, cord hemoglobin, vital signs, or capillary refill time. A study conducted in Bhopal by Hulsoore et al. also did not show much difference between LSCS and NVD. This was a larger sample size and looked at babies for the first 30 min of life after delivery. This recent study also observed no significant difference in the SpO_2_ in normal vaginal deliveries and cesarean deliveries (95% CI = 74.8612–94.8359 in NVD and 77.1231–94.6912 in LSCS] and a correlation between NVD and LSCS being significant (*r* = 0.997 at 0.01 level), with a linear trend in both. This study also showed that the SpO_2_ rise, though faster in LSCS than NVD, in both cases took 20 min of time to reach a saturation level of 90%, after which it stabilized to ≥ 90% for the next 10 min of study [21]. An interesting study on many newborns showed that LSCS babies had a higher saturation than NVD. However, this finding cannot be compared with our study as these saturation values were taken from 2 to 24 h after birth, whereas the current study focuses on the first 10 min after birth [22]. Although this study showed the effect after 2 h, a recent study from Nepal showed significantly higher SpO_2_ levels in neonates born by LSCS compared with neonates born by NVD at every point of time in the study. The study tracked saturation values from birth till 30 min after birth in 98 neonates, with 49 in each group [23].

In contrast, a host of other studies show that neonates born by LSCS have a lower risk of saturation and lower saturation compared with vaginal delivery [10, 14, 24–28]. A recent one published by Bancalari et al. from Chile showed lower saturation values in LSCS‐delivered neonates [28]. They studied 324 healthy‐term newborns, of which 160 were born vaginally and 164 by cesarean section. The SpO_2_ increased progressively from Minute 1 (58.7%) to Minute 10 (94.5%). Newborns delivered vaginally had a significantly higher SpO_2_ until Minute 10 after birth than those born by cesarean section (*p* < 0.001). Although recently published, it was conducted in 2012 and followed the older 2010 guidelines of neonatal resuscitation, with all neonates being more than 37 weeks, and a record was made of the postductal pO_2_. Similar to our study, Rabi et al. included newborns > 35 weeks of gestation. A total of 115 newborns were studied. On average, newborns delivered by LSCS had a 3% lower SpO_2_ than infants delivered by NVD (95% confidence interval [CI] = −5.8 to −0.7; *p* = 0.01). Infants born by LSCS took longer (risk ratio, 1.79) to reach a stable SpO_2_ > 85% (95% CI = 1.02–3.14; *p* = 0.04). At 5 min of age, median SpO_2_ values (interquartile range) were 87% (80%–95%) for infants delivered by NVD and 81% (75%–83%) for those delivered through LSCS. The median SpO_2_ did not reach 90% until 8 min of age in either of the two groups [14]. This study was done in 2004–2005 and followed older resuscitation guidelines. The study utilized by the 2010 NRP algorithm of the AHA was conducted by Dawson et al., which studied 468 infants, and three different investigators collected data [15]. This sample of neonates had both term and preterm infants; hence, the saturation limits are currently used for all newborns. In the first 5 min after birth, infants born through LSCS had significantly lower saturation levels than those delivered by NVD. This study was conducted before the significant changes in the algorithm that happened in 2010. The Masimo pulse oximeter with 2‐s averaging, set at maximal sensitivity, was the device used in this study, which we have used in our study. This is significant as the pulse oximeter was based on newer adaptations and technology, which minimized differences due to movement, shock, etc. In another larger study from Turkey, Zubarioglu et al. tracked 141 neonates after birth for 15 min. The time to reach > 90% preductal SpO2 level was 6.9 ± 2.8 in NVD versus 8.0 ± 2.8 compared with our study, 270 s versus 300 s for LSCS [25]. In the study by Mariani et al., neonates were mainly term with GA more than 37 weeks, emergency LSCS were excluded, but they also found that SpO_2_ levels were lower in babies delivered by LSCS as compared with NVD. The mean time taken to have a preductal SpO_2_ level of 90% was 5.2 min (1.5) in vaginal deliveries and 6.3 min (2.4) after LSCS (*p* < 0.05) [10]. In the current study, 80% of NVD neonates reached a SpO_2_ > 90% at 6 min versus 7 min in LSCS (*p* < 0.02). A recent study by Bhargava et al. from Jaipur also noted that NVD babies had higher saturation than LSCS neonates [29]. The mean SpO_2_ levels in their series were 85.4% (±6.0), 90.8% (±4.9), 94.05% (±3.8), 95.7% (±3.4), 96.7% (±2.54), and 97.4% (±2.1) at 5, 10, 15, 20, 25, and 30 min of life, respectively. Mean SpO_2_ at 10 min after birth in NVD was 91.8% (±5.1) and 89.9% (±4.48) in CS (*p* = 0.005). The mean duration for SpO_2_ to rise up to > 90% was 9.13 min (±4.9) in NVD and 12.31 min (±5.5) in CS (*p* < 0.001). This study measured levels until 30 min after birth, whereas we restricted ourselves to 10 min and showed differences at each time point, with NVD having slightly higher saturations than LSCS newborns. A multiple linear regression analysis done by Bhargava et al. showed that when SpO_2_ at 10 min was taken as a dependent variable, mode of delivery and birthweight are significant independent predictors of SpO_2_ at 10 min of life in newborns, where birth weight led to a negative impact (*β* = −0.156, *p* < 0.031), whereas NVD had a positive impact (*β* = 0.201, *p* < 0.004). Another study conducted in India showed similar findings. At 1 min of life, NVD neonates had a higher SpO_2_ range as compared with those delivered via LSCS (*p* value < 0.001). Similarly, there was a significant difference in saturation between the two groups at 5 min of life. However, at 10 min of life, the difference in the range of saturation was not much higher but was still significant (*p* value < 0.001) [30]. In a study conducted in Taiwan, where 130 full‐term neonates were monitored for the first 10 min, akin to the current study, it was found that the NVD neonates had slightly higher saturations than the LSCS neonates, but there was no significant difference after 5 min after birth [31].

In many of the studies done previously, there are saturation readings for preductal and postductal measurements, but we have not done postductal measurements as they are no longer of interest to science in this area, as preductal measurement at birth has become standard of care since 2010 onwards [32–34].

In the study by Bancalari et al., HR was also measured along with SpO_2_. Still, it was derived from the saturation monitor, unlike the current study, where we used a stethoscope to measure HR at the precordium, which was not dependent on the functioning of the saturation monitor. There were statistically significant differences found in the first 2 min postpartum with respect to the HR between the two groups, with higher average values observed in vaginal deliveries. This difference remained significant but became closer to each other till the 10 min, after which there was no difference between the two modes of delivery. On the other hand, despite the difference in saturation, our study did not show any difference between the two modalities. It is difficult to explain this, and since we used a stethoscope, our measurement may not be of high quality. Ideally, an ECG monitor should have been used as recommended in the 2015 Neonatal Resuscitation Guidelines [35, 36]. This study also showed that even in normal transition, few neonates have HR below 100 at 5 min in NVD and at 7 min in LSCS [28]. Most studies that measured HR used early cord clamping, whereas we practiced delayed cord clamping by 60 s. This avoids the depressor reflex in the Bancalari study, which may contribute to registering a higher HR in the first 3 min postnatal. The current study did not have much HR below 100 at any point in time. This is unlike Dawson et al., who showed that until around 2 min of age (the sample had both premature and term babies), less than 14% of neonates had HR less than 100 beats per minute [37]. However, at 1 min, half of these infants had an HR < 100 bpm, and 17% had an HR < 60 bpm. In this study, Dawson et al. also showed that the neonates born by NVD had a statistically significantly higher HR at every minute until about 10 min [15]. In an older study by Gonzales and Salirrosas, it was shown that neonates born by NVD had a significantly higher HR than those born by LSCS from 1 to 5 min after birth (*p* < 0.02 to *p* < 0.05) [38]. This study was done on 380 newborns in Lima, Peru, in 1998 and included even smaller infants greater than 28 weeks of GA, whereas the current study had newborns more than 35 weeks of GA. Also, these infants received oxygen during the delivery process, which is no longer the standard of care after 2010.

As part of monitoring the vitals, we measured the temperature of newborns in both delivery methods. While we have not found much difference between the two methods, this is not generalizable as the neonate′s temperature depends on the mother′s temperature and the ambient temperature of the delivery room or operating theater. Studies have shown that ensuring higher ambient temperatures in operating theaters or delivery rooms reduces newborns′ lower temperatures [39–41].

Unlike similar studies in India, we have used delayed cord clamping and placed the newborn on the mother′s abdomen, which has not been detailed in other studies. We could not use ECG for HR monitoring, the world standard for the transition of care. Intact cord resuscitation may be the standard of care in the coming years, and hence the relevance of this data may be less if it becomes a standard of care.

We conclude that the three physiological parameters we studied following the delivery of the newborn were not much different. There is a slightly higher saturation in the NVD group as compared with LSCS, with the NVD group achieving 90% saturation earlier. The HR and Temperature measurements between the two groups were comparable.

## Funding

No funding was received for this manuscript.

## Disclosure

This abstract was presented at jENS 2021, the 4th Congress of joint European Neonatal Societies on 17th September 2021 as a poster presentation in the resuscitation section. The presentation title was: Physiological Transition of Neonates Born Through LSCS as Compared to Normal Delivery.

## Conflicts of Interest

The authors declare no conflicts of interest.

## Data Availability

Data are available with the corresponding author on reasonable request.
